# Pectin Film Coated Pellets for Colon-targeted Delivery of Budesonide: *In-vitro/In-vivo* Evaluation in Induced Ulcerative Colitis in Rat

**Published:** 2012

**Authors:** Jaleh Varshosaz, Jaber Emami, Naser Tavakoli, Mohsen Minaiyan, Nakisa Rahmani, Farid Dorkoosh, Parvin Mahzouni

**Affiliations:** a *Department of Pharmaceutics, Faculty of Pharmacy and Novel Drug Delivery System Research Center, Isfahan University of Medical Sciences, Isfahan, Iran.*; b *Department of Pharmacology, Faculty of Pharmacy and Isfahan Pharmaceutical Sciences Research Center, Isfahan University of Medical Sciences, Isfahan, Iran.*; c *Department of Pharmaceutics, Faculty of Pharmacy, Tehran University of Medical Sciences, Tehran, Iran. *; d *Department of Clinical Pathology, Faculty of Medicine, Isfahan University of Medical Sciences, Isfahan, Iran.*

**Keywords:** Skull, Budesonide, Pellets, Colon delivery, Pectin, Film coating, TNBS induced colitis

## Abstract

The main objective of this study was to prepare colon-specific pellets of budesonide, using pectin as film coating. Pellet cores of budesonide were prepared by extrusion / spheronization technique. Pectin, in different ratios was combined with Eudragit RS30D, Eudragit NE30D or Surelease to produce film coating. The dissolution profiles of pectin coated pellets were investigated in pH of 1.2 (2 h), pH of 7.4 (4 h) and pH of 6.8 in the absence as well as presence of rat cecal contents (18 h). Finally the selected formulation was evaluated on trinitrobenzenesulfonic acid (TNBS) induced ulcerative colitis in rat model, in comparison with conventional UC treatments. The dissolution profiles of pectin coated pellets showed that the release of budesonide in presence of rat cecal content depended on adjuvant polymer, the ratio of pectin to polymer and film thickness. Coated pellets, prepared out of pectin and Surelease at a ratio of 1:3 at coating level of 35% (w/w), could increase budesonide release statistically in presence of rat cecal content, while they released no drug in pH of 1.2 and 7.4. Animal experiments revealed the therapeutic efficacy of pectin/Surelease-coated pellets of budesonide in alleviating the conditions of TNBS-induced colitis model as reflected by weight gain, as well as improvement of clinical, macroscopic and microscopic parameters of induced colitis. This confirmed the ability of the optimized formulation for targeted drug delivery of budesonide to colon.

## Introduction

In recent years, budesonide, a second generation glucocorticoid is approved as a standard drug for active inflammatory bowel disease (IBD) including ulcerative colitis (UC) and Crohn’s disease (CD) (1). It has the highest affinity for glucocorticoid receptor as compared to classic steroids (hydrocortisone, prednisolone, dexamethasone) (2). Currently, it is available either as oral pH-controlled-ileal-release formulation that targets the distal ileum and right-sided colonic region in Crohn’s disease, or as enema and foam for the treatment of left-sided UC (1, 3). It has been revealed that topical budesonide has no systemic effects due to high first pass effect and has a similar efficacy compared to classic topical steroids and mesalazine (5-ASA) with a better safety profile (4, 5). However, as a general principle, the acceptance and compliance of topical preparations such as enema is low and local delivery of drugs in the colon after oral administration may lead to better efficacy/side effect profiles and may improve patient›s compliance (6). In the light of this information, developing new oral formulations with an improved potential for targeting the delivery of budesonide to the colon seems essential. Among currently available oral colon delivery approaches, enzymatically controlled delivery systems containing polysaccharides (*e.g*. pectin, chitosan, xanthan gum, dextran, guar gum and inulin) are widely used to improve the specificity of drug release to colon (7). These systems are able to pass unaffected through the upper part of the gastrointestinal tract (GIT), showing biodegradability only in the colonic environment, due to the anaerobic microflora resident in this region (8). The most favorable property of these materials is that they are already approved as pharmaceutical excipients (9). Among these polysaccharides, pectin has been widely evaluated as a colon specific drug delivery entity (10). It can be broken down by pectinase enzymes produced by anaerobic bacteria of the colon and can control drug release by this principle (11). Together with other properties of pectin, such as the pH-sensitivity and film forming ability, it allows pectin-based drug delivery systems to be reliable and reproducible for colon-specific drug delivery (12).

However, the main obstacle of pectin is its high solubility and swelling properties in aqueous media, which can lead to an undesirable premature release of drugs during the transit through the upper GIT. Many approaches have been evaluated to overcome the dissolution of pectin in upper GIT, including chemical modification and combination with insoluble polymers (12). Chemical modification involves one or more additional processing steps that could affect physical and digestive properties. However, in combination with water insoluble polymers, it is thought to reach the colon intact where colonic bacteria degrade pectin resulting in increased solubility or porosity of the coating and enhanced drug release. The only problem associated with polymeric mixtures of pectin is to find the appropriate balance between hydrophobicity and hydrophilicity, which prevents drug release in upper part of the GIT, but at the same time, permit enzyme access to the polysaccharide substrate and ensure drug release at an adequate rate in the colon. Pectin has been used in numerous approaches such as pectin/ethylcellulose film coatings (13), pectin/Eudragit RL /NE film coatings (14), mixed films of pectin/chitosan/Eudragit RS (15) and mixed films of pectin/chitosan/HPMC (16). The main objective of this study was to develop, optimize and evaluate *in-vitro / in-vivo *pectin coated pellets of budesonide prepared by extrusion-spheronization technology for the treatment of UC. The optimized formulation was examined for its *in-vivo *targeting potential using 2,4,6-trinitrobenzenesulfonic acid (TNBS)-induced colitis in a rat model.

## Experimental

Budesonide was obtained as a gift sample from Astra Zeneca (UK). Ethylcellulose was used in the form of Surelease (E-7-19040, 25% solids) and was a gift from Colorcon Inc. (UK). Eudragit RS30D and Eudragit NE30D were kindly donated by Rohm Pharma (Germany). Pectin USP was gift from CPKelco (Netherlands). The microcrystalline cellulose grades (Avicel PH 101 and Avicel RC581) from FMC (Ireland), Hypermelose (HPMC 6 cps) from Shin-Etsu Chemical Co. (Japan), lactose monohydrate 200 from Meggle (Germany), talc and triethyl citrate were obtained from Kirsch Pharma (Germany), and citric acid from Kimya Gharb Gostar Chemical Co. (Iran). Trinitrobenzenesulfonic acid (TNBS) and prednisolone were purchased from Sigma Chemical Co. (St Louis, MO, USA) and were used as received. All other materials used were of analytical reagent grade and purchased from Merck Chemical Company (Darmstadt, Germany). 

*Preparation of site-specific release budesonide pellets*

*Preparation of budesonide core pellets*

Pellet cores containing budesonide (1.5% w/w), Avicel PH 101 (6% w/w), Avicel RC581 (24% w/w) and lactose (68.5% w/w) were prepared through extrusion-spheronization (extruder model 20 and spheronizer model 250, Caleva, UK). Distilled water was used as granulating liquid. They were dried at room temperature for 24 h. Pellets with the size range of 840-1000 μm were used for subsequent coating.

*Preparation of coated pellets*

Pectin (2% w/w) was first dispersed in purified water and added to the aqueous dispersions of polymers with the ratios of 1:2, 1:3, 1:4 (w/w) and stirred for 2 h prior to coating. Talc was used as antiadherent for Eudragit aqueous dispersions (50% talc based on dry polymer weight). The Eudragit RS30D aqueous dispersion also had triethyl citrate (TEC) as plastisizer (25% based on dry polymer weight). The pellet cores were coated in a FL-Mini coater, top spray fluidized bed coater (VECTOR Corporation, Japan) until a weight gain of 15, 20, 25, 30 and 35% (w/w) was achieved. A subcoat of HPMC was applied to the pellets that consisted of HPMC (5.71%, w/w), citric acid (0.2%, w/w), TEC (1.71%, w/w), talc (2.65%, w/w) and water (89.73%, w/w). The coating conditions are shown in Table 1.

**Table 1:** Operating conditions for the coating of budesonide pellets

**Condition**	**Formulations**			
	**HPMC**	**Surelease**	**Eudragit NE 30 D**	**Eudragit RS 30 D**
Nozzle diameter (mm)	1	1	1	1
Spray rate (g/min)	0.4	1	2	2
Inlet air temperature (°C)	70	60	30	40
Curing temperature (°C)	25	40	40	60
Curing time (h)	24	24	24	24

*In-vitro drug release studies*

Pellets containing 3 mg budesonide were used for *in-vitro *release studies. The USP paddle method at 37 + 0.5°C, 50 rpm were used to determine dissolution profile of the coated pellets using three consecutive media: HCl 0.1 N (250 mL) for 2 h, phosphate buffer solution (PBS) (pH of 7.4) (250 mL) for 4 h and finally, the pellets were transferred to the flask containing PBS (pH of 6.8) (100 mL) in the presence and absence of rat cecal content (4%) for 18 h under continuous supply of CO_2 _to simulate the colon environment. In order to induce enzymes specifically act on the pectin in the cecum, male Wistar rats weighing 175-200 g were fed with 1% aqueous solution of pectin for 7 days prior to the dissolution experiments. Preparation of rat cecal content containing media was carried out according to method described elsewhere (17). Sodium lauryl sulphate (SLS) (0.5 % w/v) was added to all dissolution media to maintain a sink condition for the drug release. Dissolution samples were withdrawn after regular intervals of time to evaluate drug release. The content of budesonide in the dissolution media was analyzed using an HPLC method described below.

*Determination of drug content*

The budesonide content of the pellet cores was evaluated using accurately weighed 200 mg pellets. After completely powdering pellets in a mortar, the complete residue was transferred into a 100 mL volumetric flask, 50 mL HCl 0.1 N was added and stirred for 1 h. Then 10 mL of acetonitrile along with 6.0 mL of 0.2 mg /mL of dexamethasone solution in acetonitrile were added as internal standard to the flask and made up to volume with HCl 0.1 N. This solution was kept in the ultrasonic bath for 10 min and centrifuged for 5 min at 5000 rpm. Aliquots of the solutions were filtered and analyzed by HPLC method. 

*HPLC analysis*

Determination of budesonide in the pellets and dissolution media was done by a validated HPLC method reported previously. A Shimpack C8 column (150 mm × 4.6 mm, 5 mm particle size) maintained at ambient temperature (25°C) was used for this purpose. The mobile phase consisted of acetonitrile–monobasic potassium phosphate 0.025 M (55:45, pH 3.2) which was filtered and delivered at a flow rate of 1.0 mL/min and detected by UV detection at 244 nm. Dexamethasone was applied as an internal standard. Budesonide is an epimeric mixture of two isomers which have the same pharmacologic activity. The two isomers could be detected as a single peak under the conditions described above (18).

*Scanning electron microscope (SEM) studies*

The surface characteristics of pellets before and after coating were taken using scanning electron microscope (Philips, XL30, Philips, Eindhoven, Netherlands). Samples were gold coated using a sputter coater. The shape and surface characteristic of the pellets were observed in electron micro analyzer and photographs were taken using camera, after magnification of 45, 60, 250 and 500 ×. 

*In-vivo anti-colitic effects*

*Induction of experimental colitis*

The TNBS-induced colitis rat model was selected to evaluate the new formulation of budesonide on colonic damage. All animal experiments in the present study were performed in compliance with the guidelines of Ethics committee of Isfahan University of Medical Science. Male Wistar rats (weighing 175–200 g, 12-16 weeks old) were food fasted 24 h before the experiment and allowed food and water *ad libitum *after the administration of TNBS. Colitis was induced according to the procedure described by Morris *et al*. (19) by some modifications. Briefly, after light narcotizing with ether, the rats were catheterized 8 cm intra rectally and TNBS dissolved in ethanol (40% v/v) was slowly infused into the colon (100 mg/kg) in a total volume of 0.5 mL. Animals were then maintained in a head down position for 1-2 min to prevent leakage of the intracolonic instillate and returned to their cages. Animals with instillate leakage via the anus were excluded from the study. The same procedure was performed with the normal control group but the rats were administered with normal saline instead of TNBS.

*Grouping and treatment protocols*

The animals were randomly assigned to nine groups of rats, six in each. Group A (normal group) received 0.5 mL normal saline instead of TNBS and treated orally once daily with normal saline started 24 h after colitis induction and continued for 7 days. Group B (induced colitis control) received TNBS as mentioned previously and treated with normal saline similar to group A.

In other seven groups, colitis was induced by TNBS and treatments were made orally or rectally with one of the following drugs similar to control groups. Group C, budesonide optimized formulation (300 μg/kg/day, orally), group D, budesonide solution (300 μg/kg/day, orally), group E, budesonide uncoated pellets (300 μg/kg/day, orally), group F, placebo pellets , group G, mesalazine enema (400 mg / kg/day, rectally), group H animals were treated with budesonide enema (20 mcg/kg/day, rectally) and group I, animals treated with prednisolone (5 mg /kg/day, orally ). The pellets were administered to rats via a polyethylene canula (diameter 2 mm) with 1 mL of water.

*Assessment of tissue injury*

Twenty four h after the last dose of each drug, the rats were weighed and euthanized using ether overdose and the distal colon cut at the pubic symphysis. Severity of inflammation was quantified by evaluating body weight loss, colon to body weight ratio, percent of ulcerative area and macroscopic and microscopic indices. Using the distal colon (8 cm), 2 cm proximal to the anus, ulcer surface area (mm^2^) and the ratio of colon weight versus rat body weight (mg/g) were measured as described previously (21). Macroscopic damage was assessed througha scoring system set forth previously (22) with a slight modification as follows: 0, normal appearance; 1, erythema and inflammation without ulcer; 2, Inflammation and ulcer; 3, ulcer with necrosis. After macroscopic evaluation, biopsies of 1 cm in length of the colon containing all layers were fixed in 10% buffered formalin solution, embedded in paraffin, stained with haematoxylin and eosin (H & E) and sent for histopathological studies. The microscopic evaluation was carried out by a pathologist unaware of the study design. Inflammation severity, inflammation extent and crypt damage were the criteria that considered assessing the colon damage from the histological point of view (23). The criteria for the microscopic evaluation are shown in Table 2. 

**Table 2:** Scoring criteria for histopathological assessment of induced colitis (24).

**Scoring parameter**	**Score definition**
Inflammation severity	0: None 1: Mild 2: Moderate 3: Severe
Inflammation extent	0: None 1: Mucosa 2: Mucosa and submucosa 3: Transmural
Crypt damage	0: None 1: Basal 1/3 damaged 2: Basal 2/3 damaged 3: Crypts lost, surface epithelium present 4: Crypts lost, surface epithelium lost

The scores for each category examined were calculated for each specimen in the different groups. These were then added to obtain the total score, which was then divided by the number of rat colons examined in each group to obtain the total histologic score of induced colitis for the group.

*Statistical analysis*

SPSS software version 11.5 was used for all statistical analysis. One-way analysis of variance (ANOVA) followed by a Tukey›s post hoc test was used for comparison between cumulative percentage of budesonide released at the end of each dissolution test. *In-vivo *data were expressed as mean ± SEM. Differences between mean values of parametric data; colon weight/Body weight ratio and ulcer area were analyzed using one-way analysis of variance (ANOVA) followed by a Dunnett›s post hoc. Comparison between non-parametric variables of different groups; macroscopic damage score and microscopic parameters, was performed using non-parametric Mann-Whitney U-test. A significant level of p *< *0.05 denoted significance in all cases.

## Results and Discussion

*In-vitro results*

Various colon specific delivery systems based on the colonic microflora have been used for colon targeting of budesonide including dextran tablet (24), chitosan-coated Ca-alginate microparticles (25), guar gum and khaya gum coated tablet (26), pectin compressed coated tablet (27) and chitosan-chondroitin sulfate coated tablet (28).

**Figure 1:**
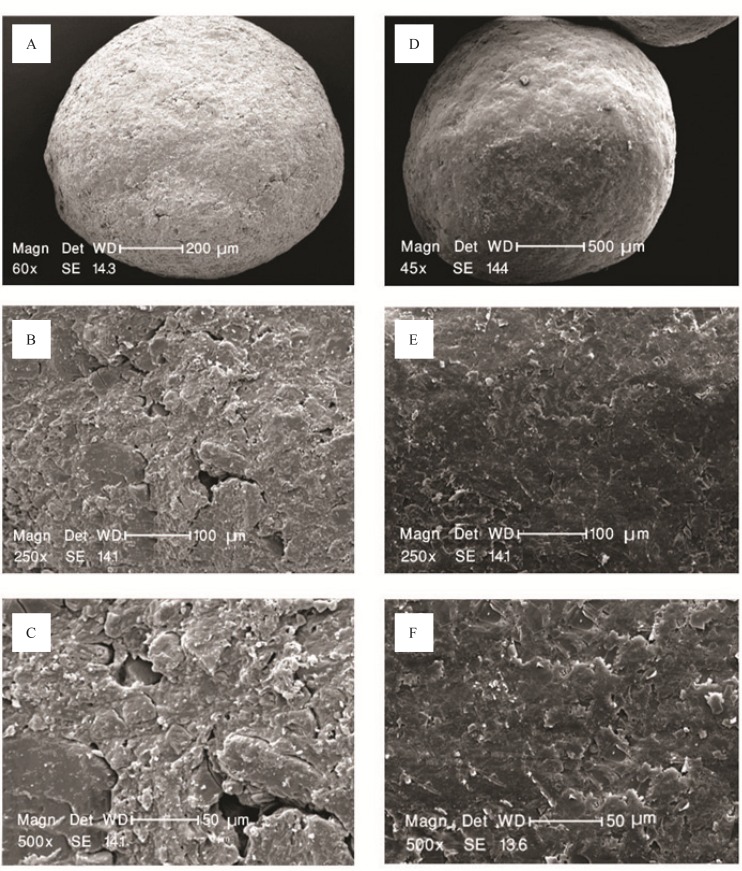
SEM pictures of the surface of budesonide pellets with (a) Magnification 60×, (b) Magnification 250×, (c) Magnification 500×, and after coating with pectin/ Surelease in ratio of 1:3, weight gain 35% with (d) magnification 45×, (e) Magnification 250×, and (f) Magnification 500×.

Budesonide pellet cores were successfully prepared by extrusion-spheronization method. The drug-loaded pellets (Figure 1 a-c) were found spherical with satisfactory physical properties.

The objective of applying a sub-coat of HPMC containing citric acid onto the budesonide loaded pellets prior to the application of main coating was to diminish any probable interaction between budesonide and coating substances especially Surelease and also smoothing the surface of the pellets prior to coating.

**Figure 2:**
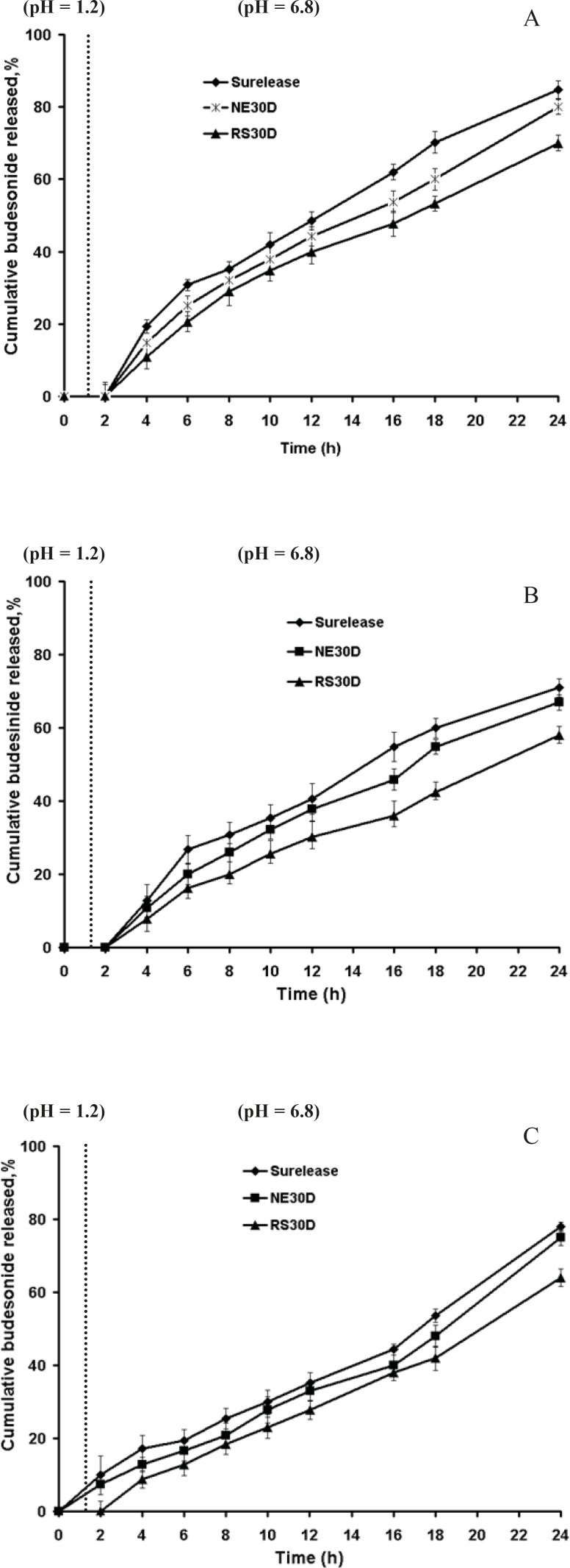
In-vitro release profiles of budesonide from pellets coated with 1:2 pectin/polymer ratio at coating level of a) 20% w/w, b) 30% w/w and c) 1:3 pectin/polymer ratio at coating level of 20%.

HPMC- coated pellets were subsequently coated with pectin in combination with each of the three polymers (1:2, 1:3, 1:4 ratios) with different coating levels. Representative budesonide release profile of pellets coated with pectin are shown in Figure 2a-c. In Figure 2a (1:2 pectin polymer ratio, gaining 20% w/w thickness) although no drug release was observed in acidic phase, due to rapid water uptake, swelling and rigid gel formation of pectin in HCl 0.1 N (pH 1.2), it was accompanied with a sudden increase in drug release in PBS (pH 6.8) in further 4 h. At this ratio, there was very little difference between the release rates of the three polymers. Figure 2b demonstrated that when the coating level was increased to 30% w/w, there was further retardation of drug release by 24 h; however there was still some drug release in PBS (pH 6.8) in further 4 h.

Figure 2c showed that film coated pellets prepared from pectin and polymer (in a 1:3 ratio) at coating level of 20% w/w, released lower drug within 24 h due to enhanced resistance of pellets. However, release of budesonide in HCl 0.1 N was seen to be higher compared to pellets coated with pectin-polymer in 1:2 ratio, being strongly apparent from the pectin-Surelease coated pellets. The poor water solubility of budesonide masked the influence of the coating composition on the drug release from Eudragit RS and NE coated pellets.

To improve budesonide release pattern, further pectin-polymer ratios of 1:3, 1:4 and higher coating levels were taken (Table 3) in coating films to observe the effects on drug release after 24 h dissolution studies. Drug release studies were conducted under pH conditions mimicking the stomach, small intestine and colon in absence and presence of rat cecal contents.

**Table 3:** Formulation of film coating compositions

**Formulation code**	**Pectin/Polymer ratio**	**Polymer type**	**%Theoretical weight gain**
F1	1:3	Surelease	30
F2	1:4	Surelease	30
F3	1:3	Surelease	35
F4	1:4	Eudragit NE	25
F5	1:3	Eudragit NE	30
F6	1:4	Eudragit NE	30
F7	1:3	Eudragit RS	25
F8	1:4	Eudragit RS	25

**Figure 3:**
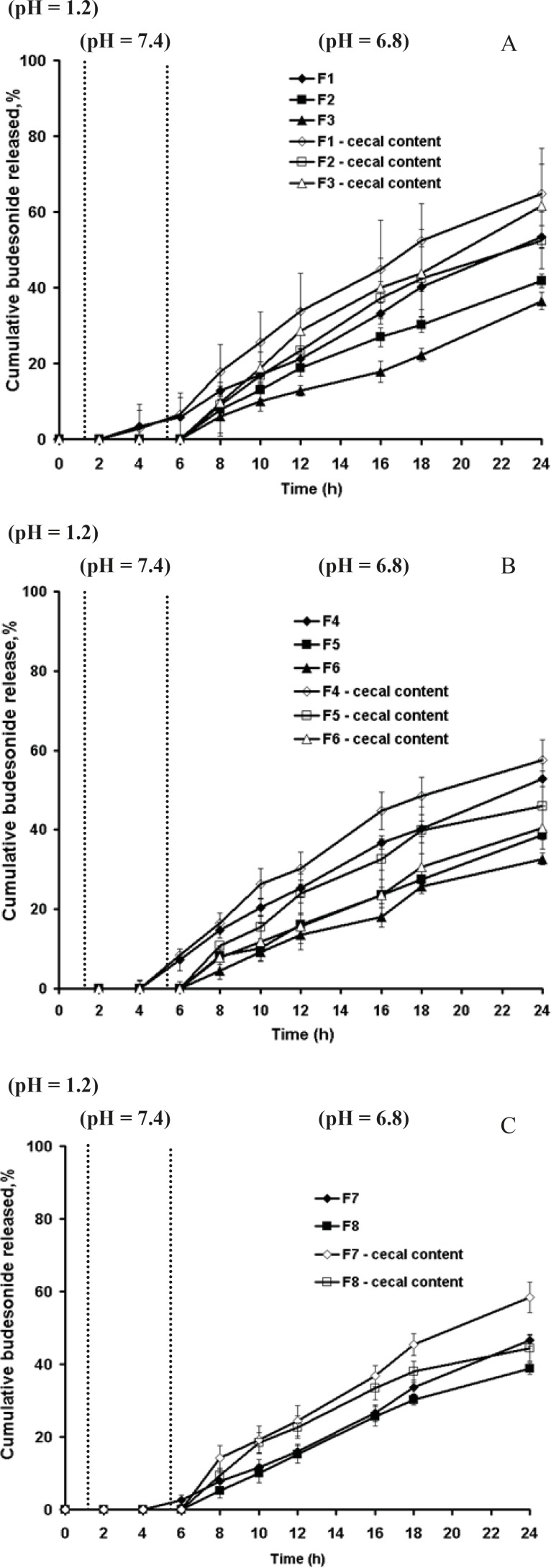
In-vitro release profiles of budesonide from pellets coated with (a) pectin/Surelease (b) pectin/Eudragit NE30D, (c) pectin/Eudragit RS30D in absence and presence of rat cecal content

Figure 3a-c showed that in absence of rat cecal content, the dissolution profiles of these formulations are characterized by an initial lag time, whose duration depends on the pectin content, the thickness of coating and permeability of the polymer. Presence of rat cecal content in the dissolution media resulted in an increase of budesonide release from all the coated pellets perhaps due to the microbial degradation of pectin in the coat film that creates aqueous channels or water filled pores that allow diffusion of drug molecules through the film coatings. However this was only significant for F1 and F3 (Surelease coated pellets) (p < 0.05) (Figure 3a).

Although Semde *et al. *(29) reported that only films of pectin and Eudragit RS30D were suitable carriers for colonic drug delivery, our studies manifested the suitability of Surelease along with Eudragit RS30D in combination with pectin for colon targeting. While Eudragit RS30D was markedly better in preventing budesonide release in gastric and small intestine simulated conditions at each film weight and ratio; Eudragit RS based film coated pellets revealed the least increased release rates in cecal-containing medium (Figure 3c). Perhaps due to the low permeability properties of Eudragit RS, incorporated pectin was not accessible enough to enzymatic degradation. In contrast, it seems that Surelease characterized by higher permeability properties as compared to Eudragit NE and Eudragit RS, had better enzymatic accessibility of pectin within the polymer structure and showed the maximum increase in budesonide release. Based on the release data obtained, the polymer type showed a stronger impact on drug release compared to coating thickness and pectin - polymer ratio parameters. Here two features must be taken into consideration; the first is the low viability of microflora *in-vitro *that affects the results of release test. While*, *the bacteria continuously produce the respective enzymes, we expect more budesonide released *in-vivo*. Another issue to note is the upper GIT motility, which creates significant mechanical strength that could induce the formation of cracks in the polymeric film coatings, ans causes much higher drug release rates. The *in-vivo *performance of these polymeric blends may be expected to be better.

The pectin coated pellets at the 1:3 ratio of pectin: Surelease at the coating level of 35% w/w (F3) was selected for the next *in-vivo *experiments on TNBS- induced UC in rat model.

*In vivo anti-colitic effects*

The effectiveness of the optimized formulation of pectin coated budesonide pellets was evaluated in comparison with budesonide solution, uncoated budesonide pellets and also standard treatments of this disease including oral prednisolone, topical budesonide and mesalazine using TNBS-induced colitis model in rats.

Severity of inflammation was quantified by evaluating body weight loss, colon to body weight ratio, percent of ulcerative area and macroscopic and microscopic indices.

The animals in the TNBS group presented a strong inflammatory reaction limited to the distal colon with ulcers, mucosal necrosis and thickening of the intestinal wall. This was associated first with anorexia and weight loss and signs of diarrhea in most animals. On day 0, body weight of rats were 180 ± 8.3 g in healthy control group (n = 6) and 182.5 ± 12.7 g in TNBS treated groups (n = 48), where there was no significant difference between them. The average weight gain in healthy control group was 28.3 ± 1.6 g, but the rats in colitis group presented significant body weight loss (34 ± 5 g) (p < 0.05). Except placebo treated group, all treatments improved the body weight of rats during treatment period in comparison with colitis group (p < 0.05) as treatment with pectin coated pellets reversed the body weight loss (11.5 ± 2.4) significantly.

As the data in Table 4 show, the colon index was significantly higher in colitis control group (12.9 ± 1.7) than that obtained in the healthy control group (3.6 ± 1.4) (p < 0.05). Increases in the colon index after TNBS administration were significantly inhibited by pectin coated pellets of budesonide. Ulcerative area also diminished in comparison with colitis control group (p < 0.05). In each case budesonide pectin coated pellets was superior to budesonide solution and uncoated pellets (p < 0.05).

**Figure 4:**
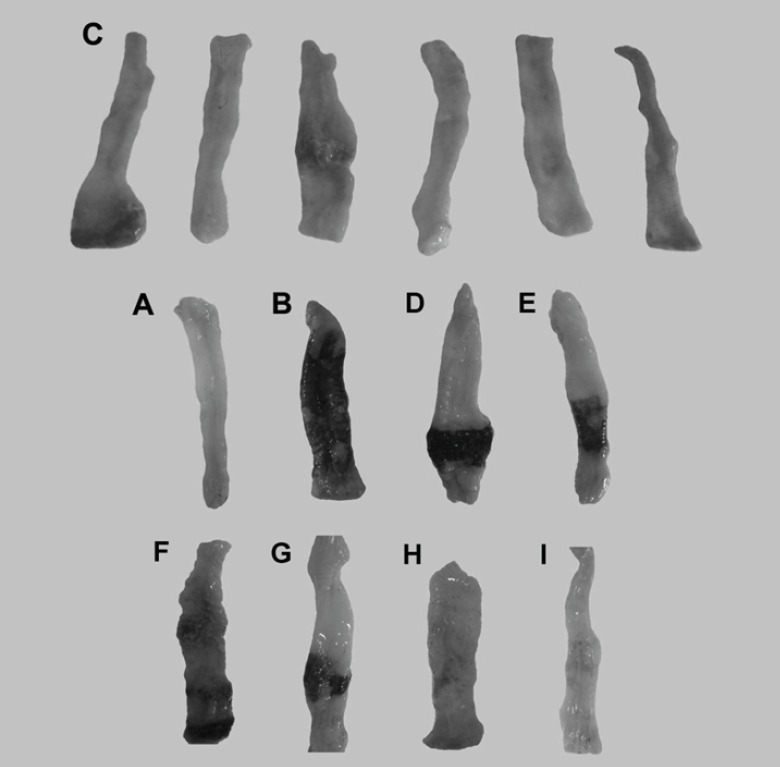
Representative photographs of macroscopic appearance of rat colonic mucosa. A = Normal control group, B= colitis control group, C= Budesonide pectin/Surelease coated pellets (300 μg/kg/day) improved TNBS–induced colitis and decreased the ulcer surface, D = Budesonide solution (300 μg/kg/day), E = Budesonide uncoated pellet (300 μg/kg/day), F = Placebo pellet group, G = Mesalazine enema (400 mg/kg/day), H = Budesonide enema (20 mcg/kg/day, rectally), I = prednisolone (5 mg /kg /day ,oral ).

When rats were treated with saline, there was no macroscopic damage (Figure 4A). In contrast, the colon in colitis control group was severely damaged, showing mucosal hyperaemia, haemorrhage, deep ulcers and necrosis (Figure 4B) (19). As reported previously budesonide solution did not lead to any improvement of ulceration and inflammation (Figure 4D) (30). This may be due to the fact that budesonide in solution form can readily be absorbed from the stomach and small intestine and could not be delivered to the colon and because budesonide has a large first pass metabolism, a very low concentration of the drug is delivered to the afflicted area. The uncoated pellets did not affect the induced damage statistically due to similar reason (Figure 4E). In contrast to budesonide solution and uncoated pellets, in the group treated with new budesonide formulation less inflammation and smaller ulcers were detected and colonic damage score decreased significantly (p < 0.05) (Figure 4C). 

**Figure 5:**
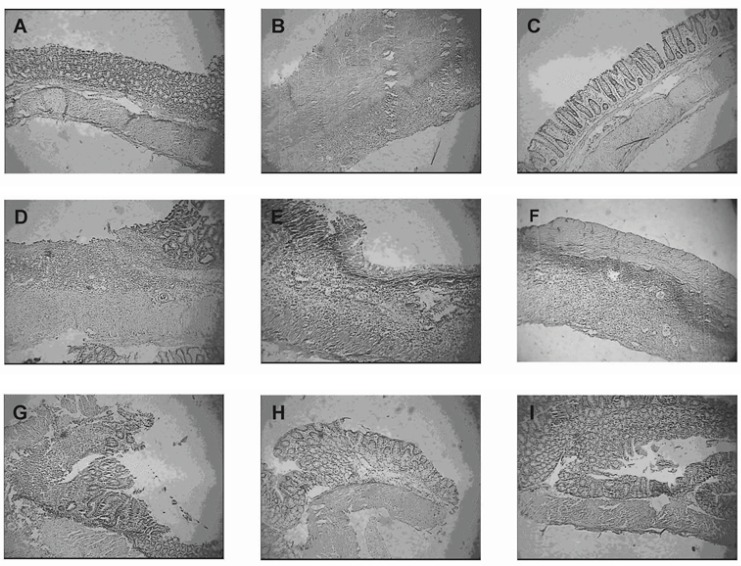
Representative photographs of histological appearance of rat colonic mucosa. A= Normal control group, B= Colitis control group, after exposure to TNBS (100 mg/kg) the colon is markedly inflamed, the mucosal wall is thickened, and there is a transmural inflammatory cell infiltration. C= Budesonide pectin/Surelease coated pellet (300 μg/kg/day) group, D= Budesonide solution group (300 μg/kg/day), E=Budesonide uncoated pellet group (300 μg/kg/day), F= Placebo pellet group, G= Mesalazine enema (400 mg / kg/day, rectally) group, H= Budesonide enema (20 mcg/kg/day, rectally) group and I= Prednisolone (5 mg /kg /day, oral) group. Hematoxylin and eosin stain with original magnification 10×.

The histological segments of treatment groups are show in Figure 5. The group treated with pectin coated budesonide pellets, showed histological improvement in comparison with colitis control group and could attenuate the total histological score of colitis (p < 0.05). The histological score was also significantly decreased in rats treated with budesonide coated pellets compared with those given budesonide oral solution and budesonide uncoated pellets (Table 5) (p < 0.05). 

**Table 4:** Data of clinical score and macroscopic evaluation of colitis in control and treatment groups after 7 days treatment

**Groups**	**A**	**B**	C _*+_	D _*_	**E**	**F**	**G**	**H**	**I**
ΔBW(g)	28.3 ± 1.6	-34 ± 5.0	11.5 ± 2.4_*+_	3.5 ± 2.7	4.2 ± 2.8	-20 ± 3.2	-5 ± 5.1	17.8 ± 1.7	9 ± 6.9
Cw /Bw (mg/g)	2.5 ± 0.7	12.9 ± 0.7	6.6 ± 0.9_*+_	9.1 ± 0.2	8.4 ± 0.2	10.7 ± 0.9	5.5 ± 0.3_*_	4.7 ± 0.4_*_	3.6 ± 0.6_*_
Ulcerative area (%)	0.0 ± 0.0	69.7 ± 5.3	28.6 ± 3.5_*+_	50.2 ± 4.1	51.8 ± 3.7	71.8 ± 3.9	40 ± 7.8_*_	24 ± 6.5_*_	13.1 ± 0.9_*_
Macroscopic score	0.0 ± 0.0	3.0 ± 0.0	2.4 ± 0.3	2.7 ± 0.2	2.7 ± 0.3	2.8 ± 0.4	2.7 ± 0.2	2.1 ± 0.4	2.0 ± 0.08

ΔBW = Body Weight change; Cw/Bw = Colon weight to body weight ratio; Ulcerative area (%) = Ulceration area /Total area of 8 cm colon; A = Normal group; B = Colitis group; C = Budesonide pectin-coated pellets (300 μg/Kg/day); D = Budesonide solution (300 μg/Kg/day); E = Budesonide uncoated pellets (300 μg/Kg/day); F = Placebo pellets; G = Mesalazine enema (400 mg/Kg/day, rectally); H = Budesonide enema (20 mcg/Kg/day, rectally); I = Prednisolone (5 mg/Kg/day, oral).

The results were expressed as mean ± SEM, (n = 6).*p < 0.05, denote significant difference vs. colitis control group; +p < 0.05 denote significant difference vs. budesonide solution, budesonide uncoated pellets.

**Table 5:** Data of histopathologic evaluation of colitis in control and treatment groups after 7 days treatment.

**Groups**	**IS**	**IE**	**CD**	**TMS**
A	0 ± 0	0 ± 0	0 ± 0	0 ± 0
B	2.8 ± 0.15	2.8 ± 0.15	3.3 ± 0.5	8.14 ± 1.3_*__+_
C	2.2 ± 0.4	2.2 ± 0.4	2.0 ± 0.6	6.0 ± 1.3
D	2.33 ± 0.30	2.16 ± 0.4	3.70 ± 0.3	7.57 ± 0.9
E	2.50 ± 0.20	2.16 ± 0.3	2.83 ± 0.8	7.0 ± 1.0
F	2.66 ± 0.20	2.66 ± 0.4	3.0 ± 0.8	7.57 ± 0.7_*_
G	2.83 ± 0.15	2.66 ± 0.2	2.28 ± 1.0	6.14 ± 1.0_*_
H	3.0 ± 0.90	2.50 ± 0.20	2.57 ± 1.1	6.85 ± 0.5_*_
I	2.16 ± 0.4	1.66 ± 0.2	1.85 ± 1.3	4.71 ± 1.0

A = Normal control; B = Colitis control; C = Budesonide Surelease coated pellets (300 μg/Kg/day); D = Budesonide solution (300 μg/Kg/day); E = Budesonide uncoated pellets (300 μg/Kg/day), F = placebo pellets; G = mesalazine enema (400 mg/Kg/day, rectally), H = budesonide enema (20 mcg/Kg/day, rectally), I = prednisolone (5 mg/Kg/day, oral). IS = inflammation Severity, IE = Inflammation Extent; CD = Crypt Damage, TMS = Total Microscopic Score. The results were expressed as mean ± SEM, (n = 6), *p < 0.05, denote significant difference vs. colitis control group, +p < 0.05, denote significant difference vs. budesonide solution, budesonide uncoated pellets

Figure 4 shows the colonic macroscopic ulceration observed in colonic segments for each group. 

The standard treatments significantly reduced all these parameters in comparison to colitis group (p < 0.05). However, the most benefit with respect to colitis severity was observed following the administration of prednisolone. In case of body weight loss, new budesonide formulation was superior to topical mesalazine (-5 ± 5.1) (p < 0.05) (Table 4). Its effect on colon index was similar to that in mesalazine enema and budesonide enema groups (p > 0.05) and diminishing effect on ulcerative area was comparable to the group treated with mesalazine enema. 

In general these data confirm that pectin coating led to enhancement of budesonide effectiveness in colitis as reflected by weight gain, as well as improvement of clinical, macroscopic and microscopic parameters of colitis. Similar studies carried out with colonic delivery systems containing budesonide, such as glucoronide prodrugs (31), azopolymer coated pellets (32), micro-encapsulated cellulosic cores (30) and pH sensitive nanospheres (33) revealed the increased efficacy of budesonide compared to oral administration of the free drug.

 The results lead us to the conclusion that in the studied experimental model, the new colonic delivery system significantly improved the efficacy of budesonide in the healing of induced colitis in rats. The described system may therefore be useful for clinical treatment of human colonic IBD.

## Conclusion

As an overall conclusion, pectin film coatings are regarded as being suitable for preventing the release of budesonide in the upper parts of GIT with no need to any other enteric coating. However the pectin based coating performance in the colon was shown to be dependent on the adjunct polymer used, the ratio of pectin in the film as well as film thickness. Therefore the pellets coated with 1:3 ratio of pectin/Surelease at a 35% w/w weight gain could release more than 60% of budesonide in presence of rat cecal contents while released no drug in pH 1.2 and pH 7.4. Although the complete drug release was not achieved in the release experiment perhaps due to low viability of the colonic microflora used in our experiment, the results of *in-vivo *test allow us to conclude that the new formulation of budesonide may be useful for further pharmacokinetic evaluation.
